# The effect of cardioplegic supplementation with sildenafil on cardiac energetics in a piglet model of cardiopulmonary bypass and cardioplegic arrest with warm or cold cardioplegia

**DOI:** 10.3389/fcvm.2023.1194645

**Published:** 2023-06-07

**Authors:** Katie L. Skeffington, Eltayeb Mohamed Ahmed, Filippo Rapetto, Guillaume Chanoit, Andrew R. Bond, Antonella Vardeu, Mohamed T. Ghorbel, M-Saadeh Suleiman, Massimo Caputo

**Affiliations:** ^1^ Bristol Heart Institute, University of Bristol, Research Floor Level 7, Bristol Royal Infirmary, Bristol, United Kingdom; ^2^ Department of Cardiac Surgery, Bristol Royal Infirmary, Bristol, United Kingdom; ^3^ Langford Vets, University of Bristol, Langford, Bristol, United Kingdom

**Keywords:** cardiac surgery, cardioprotection, ischaemia and reperfusion injury, congenital heart disease, sildenafil, cardiopulmonary bypass

## Abstract

Cardioplegic cardioprotection strategies used during paediatric open-heart surgery remain suboptimal. Sildenafil, a phosphodiesterase 5 (PDE-5) inhibitor, has been shown to be cardioprotective against ischemia/reperfusion injury in a variety of experimental models and this study therefore tested the efficacy of supplementation of cardioplegia with sildenafil in a piglet model of cardiopulmonary bypass and arrest, using both cold and warm cardioplegia protocols. Piglets were anaesthetized and placed on coronary pulmonary bypass (CPB), the aorta cross-clamped and the hearts arrested for 60 min with cardioplegia with or without sildenafil (10 nM). Twenty minutes after removal of cross clamp (reperfusion), attempts were made to wean the pigs from CPB. Termination was carried out after 60 min reperfusion. Throughout the protocol blood and left ventricular tissue samples were taken for analysis of selected metabolites (using HPLC) and troponin I. In both the cold and warm cardioplegia protocols there was evidence that sildenafil supplementation resulted in faster recovery of ATP levels, improved energy charge (a measure of metabolic flux) and altered release of hypoxanthine and inosine, two purine catabolites. There was no effect on troponin release within the studied short timeframe. In conclusion, sildenafil supplementation of cardioplegia resulted in improved cardiac energetics in a translational animal model of paediatric CPB surgery.

## 1. Introduction

Medical advances have facilitated significant improvements in the success of operations performed to repair congenital heart malformations in paediatric patients (1). During many open-heart surgeries, hyperkalemic cardioplegia is administered to the heart, resulting in depolarized cardiac arrest. This not only creates a still, bloodless field for the surgeon to operate in but also reduces myocardial metabolism, thus delaying the deleterious effects of ischaemic reperfusion (I/R) injury (2). However, despite the fact it has been known for many years that paediatric hearts have physiological differences compared to adult hearts which affect their susceptibility to I/R injury (3–9), cardioplegic strategies have mostly been extrapolated from adult protocols. Importantly, there is now evidence that the myocardial protection afforded is significantly less effective in paediatric patients compared to adults (10–14).

In recent years, interest has grown in the use of phosphodiesterase 5 (PDE-5) inhibitors, such as sildenafil, in protection against I/R injury. Several animal studies have demonstrated that administration of sildenafil either to cardiomyocytes, isolated hearts or *in vivo,* can result in reduced infarct size, improved cardiac function, reduced fibrosis and lower mortality following I/R injury (15–22). Sildenafil is known to dilate the coronary vasculature (23), however the effectiveness of sildenafil mediated protection in isolated cardiomyocytes (17, 18) suggests that there must also be direct effects on the myocardium. Several studies have focused on protein kinase G (PKG) signalling pathways, which are known to play an important role in cardio-protection (24). Sildenafil administration has been demonstrated to result in increased PKG activity (18) as well as alterations to proteins downstream of PKG including increased BCL-2 expression (16–18, 25, 26), phosphorylation of ERK1/2 and GSK3ß (16, 18), opening of mitochondrial *K*_ATP_ channels (19, 20) and inhibition of the opening of mitochondrial permeability transition pores (MPTPs) (27). Pharmacological inhibition at various points of this pathway has been demonstrated to prevent the cardio-protective effects of sildenafil administration (16, 18–20, 28). Other studies have demonstrated that sildenafil mediated cardio-protection also involves protein kinase C (PKC) translocation (15, 28) and increased activity of eNOS and iNOS enzymes (16, 17, 26, 29).

Unpublished work from our own laboratory has demonstrated in both rats and pigs that the expression of PDE5 is higher in neonatal animals than adult animals, suggesting that PDE5 may be an effective target for cardioprotection during paediatric surgery. Sildenafil is already used clinically in paediatric patients for the treatment of pulmonary arterial hypertension (30–32), so the drug is known to be tolerated by this group of patients. The current study therefore aimed to test the cardioprotective efficacy of supplementation of cardioplegia with a clinically relevant dose of sildenafil in a piglet model of cardiopulmonary bypass (CPB) and cardioplegic arrest. Current clinical practice favours the use of cold blood cardioplegia for paediatric open-heart surgery, however there is also increasing interest in the use of normothermic CPB for these patients (33–35). For this reason, we tested the efficacy of sildenafil in both cold and warm cardioplegia protocols.

## 2. Methods

### 2.1. Anaesthesia

All procedures were approved by the University of Bristol Animal Welfare and Ethical Review Body and were performed in accordance with the UK Animals (Scientific Procedures) Act 1986. Anaesthesia was standardised between all piglets (38 female Landrace piglets, around 12 kg in weight—Table 1). The piglets were fasted overnight and sedated using an intramuscular injection of ketamine (15 mg/kg), morphine (0.2 mg/kg) and midazolam (0.3 mg/kg) before induction of anaesthesia (propofol 2 mg/kg, i.v.). The piglets were then intubated using a cuffed endotracheal tube and mechanically ventilated; anaesthesia was maintained using isoflurane in an O_2_/air mix. Fentanyl (10 µg/kg/h, i.v.) and pancuronium (0.1 mg/kg/h i.v.) were continuously infused to provide analgesia and muscle relaxation respectively. Standard physiological monitoring was performed throughout as was monitoring of neuromuscular blockade using train-of-four stimulation.

**Table 1:** Body weights of piglets in each treatment group.

	Body weight (kg)	
	Cold cardioplegia	Warm cardioplegia
Control	11.4 ± 0.6 (*n* = 7)	12.1 ± 0.4 (*n* = 12)
Sildenafil	12.8 ± 0.6 (*n* = 8)	12.1 ± 0.6 (*n* = 11)

There was no significant difference between the control and sildenafil groups in each experiment (two-tailed, unpaired Student's *t*-test).

### 2.2. Cardiopulmonary bypass

A median sternotomy was performed to expose the heart and great vessels and the thymus was partially excised. Heparin (400 IU/kg, i.v.) was administered, the right atrium and ascending aorta were cannulated and cardiopulmonary bypass (CPB) commenced. A cannula with Y adaptor was inserted into the ascending aorta to administer blood cardioplegia and act as a supra-aortic vent. An aortic cross clamp was applied and cardioplegic arrest was induced and maintained for 60 min. The blood pressure on cardiopulmonary bypass was maintained around 40–50 mmHg, using bolus doses of metaraminol as needed. Cardioplegic protocols followed clinical practice used in Bristol Children's Hospital.

### 2.3. Cardioplegia

In the cold cardioplegia group, arrest was induced by administration of 110 ml/min/m^2^ cold blood cardioplegia for four minutes, with blood: cardioplegia (sterile concentrate for cardioplegia infusion, Martindale Pharmaceuticals, Romford, UK) ratio of 4:1. Arrest was maintained after 30 min by a maintenance dose (110 ml/min/m^2^) for two minutes, with additional maintenance doses given if the heart resumed activity between doses of cardioplegia. For all doses the concentration of both MgCl_2_ and KCl in the cardioplegia arriving at the heart was 8 mmol/L. Cardiac temperature was monitored with a flexible temperature probe inserted into the left ventricular wall to monitor the cooling of the heart.

In the warm cardioplegia group, arrest was induced by administration of 205 ml/min/m^2^ warm blood cardioplegia over two minutes, maintained every twenty minutes with a maintenance dose (130 ml/min/m^2^) for one minute, with additional maintenance doses given if the heart resumed activity between doses of cardioplegia. For all doses the concentration of both MgCl_2_ and KCl in the cardioplegia arriving at the heart was 18.7 mmol/L.

For both the warm and cold protocols, the cardioplegia solution arriving at the heart contained a sildenafil (Revatio, Pfizer, New York) concentration of either 0 or 10 nmol/L, a concentration which is well below the dosage used clinically in paediatric patients for the treatment of pulmonary arterial hypertension and reported therapeutic plasma concentrations (36, 37). The surgeon, anaesthetist and perfusionist were all blinded as to whether the cardioplegia contained sildenafil or not (but it was not possible to blind whether the cold or warm cardioplegia protocol was being used).

### 2.4. Cardiac reperfusion

Following 60 min of arrest, the aortic cross-clamp was released. If fibrillation occurred, up to 5 electrical shocks were given to induce sinus rhythm. Ventilation was then resumed, and the heart weaned off cardiopulmonary bypass 20 min after releasing the cross clamp. Protamine (5 mg/kg) was administered to reverse heparinisation and the animal swiftly decannulated. Following a total of one hour of reperfusion, the experiment was be terminated via an injection of pentobarbitone (0.1 mg/kg i.v.). If the pig could not be weaned off bypass it remained on bypass until termination.

### 2.5. Sample collection

Arterial blood samples were collected immediately before CPB commenced, immediately before cardioplegia was administered, immediately before the cross-clamp was removed, at 1, 10, 20 and 60 min reperfusion. Samples were collected by the perfusionist whilst the pig was on CPB and by the anesthetist at all other timepoints. Blood was spun immediately (1,000 g for 10 min at 4°C) and the plasma stored at −80°C. Cardiac tissue biopsies were collected from the left ventricle by the surgeon at four timepoints (immediately before CPB, immediately before release of the aortic cross-clamp, at 20 min reperfusion and at termination) using a trucut needle. Biopsies were immediately flash frozen in liquid nitrogen before storage at −80°C. Arterial blood gases (EPOC portable blood gas machine) were collected throughout the protocol by the anesthetist and perfusionist as considered necessary for clinical practice. For analysis, the blood gases were grouped into three time periods (before CPB, whilst the aortic cross-clamp was on and after cross-clamp release). If a pig had more than one blood gas measured during a time period, an average was calculated.

### 2.6. Processing of tissue biopsies

The tissue biopsies were crushed whilst still frozen in liquid nitrogen using a pestle and mortar. The resulting tissue powder was immediately transferred into a weighed LP3 tube containing 4.8% perchloric acid at 4°C. Wet tissue weight was calculated by reweighing the tubes following addition of the tissue. The mixture was vortexed and centrifuged (4,000 g, 10 min, 4°C) before the supernatant was neutralized by mixing with 0.44 M K_2_CO_3._ The volumes used were chosen to generate a neutral pH, with the volumes noted for subsequent calculation of the dilution factor. The mixture was then re-centrifuged before the supernatant was collected, aliquoted and stored at −20°C freezer for HPLC analysis.

### 2.7. HPLC

High-performance liquid chromatography (HPLC) was used to separate and identify metabolites as reported previously (11, 38–40). The signals generated from HPLC were displayed as chromatograms using (OpenLab Software, Aligent Technologies, USA). Peaks of each metabolite in the sample chromatograms were identified using standards of known concentration. Data analysis was completed by measuring the area under the curve for each peak, and the values were corrected to account for the dilution factor and the weight of each biopsy to produce the concentration of each metabolite. Data for one pig in the sildenafil group of the warm cardioplegia experiment was removed due to having an extremely high baseline value of troponin (∼13 ng/ml). Energy charge for adenylates has been used as a measure of metabolic flux rate (41) and is calculated as follows:

$$\mathrm{Energy}\;\mathrm{charge}\;=\frac{\mathrm{ATP}+0.5\;\mathrm{ADP}}{\mathrm{ATP}+\mathrm{ADP}+\mathrm{AMP}}$$

### 2.8. Troponin

Concentrations of troponin I were measured in the pig plasma using a commercially available Elisa kit (CTNI-9-HSP, Life Diagnostics, West Chester, USA). In brief, samples were diluted and incubated in a 96 well plate (150 rpm, room temperature) with two antibodies (one for solid phase immobilization and one for HRP conjugation). Wells were washed to remove excess HRP conjugate, and TMB added. In the presence of cTNI a blue colour develops, which was converted into yellow by addition of stop solution. Absorbance was measured at 450 nm, and concentrations calculated by comparison to a standard curve.

### 2.9. Statistics

For comparisons between two groups, the data was first tested for normality using the Shipiro–Wilk test. Normal data was analyzed with unpaired, 2-tailed *t*-tests whilst non-normal data was analyzed with Mann–Whitney *U* test. Categorical data was analysed with Chi squared tests. Levels of metabolites and troponin were analyzed by 2-way mixed model ANOVA with one repeated measures factor (time) and one between subjects factor (treatment group). A one-way ANOVA with post-hoc Dunnett's test where appropriate was also performed for each treatment group to compare which timepoints were significantly different from baseline. All statistics were performed in SPSS (Version 28, IBM Analytics, New York, USA) and significance was accepted when *p* < 0.05.

## 3. Results

### 3.1. Animal characteristics

There was no significant difference between the body weights of piglets used in the control and sildenafil groups in either the cold cardioplegia or the warm cardioplegia experiments (Table 1). Measurements of cardiac temperature in the cold cardioplegia group confirms that the cardiac tissue was effectively cooled by administration of the cardioplegia, and that there was no difference in the temperatures obtained between the control and sildenafil groups (Table 2). Blood gases throughout the protocol for each group of animals are shown in Supplementary Tables S1, S2.

**Table 2:** Cardiac temperature in the cold cardioplegia experiment.

	Time since start of ischemia (min)	Cardiac temperature (°C)	
		Control (*n* = 6)	Sildenafil (*n* = 7)
Before cardioplegia	0	36.3 ± 0.6	36.0 ± 0.3
After initial cardioplegia dose	4	15.7 ± 1.8^a^	12.2 ± 1.5^a^
Before maintenance cardioplegia dose	30	30.0 ± 1.0^a^	28.5 ± 1.1^a^
After maintenance cardioplegia dose	32	23.4 ± 1.3^a^	18.1 ± 2.1^a^
At the end of ischaemia	60	30.8 ± 1.3^a^	30.8 ± 0.8^a^

There were no significant differences between the control and sildenafil groups at any timepoint (two-tailed, unpaired Student's *t*-test or Mann–Whitney *U* test as appropriate).

^a^ Represents a significant difference vs. the ‘Before cardioplegia’ timepoint in the same treatment group (one-way ANOVA with *post-hoc* Dunnett's test.

#### 3.2. Sildenafil, cardioplegic arrest and weaning from CPB

**Figure 1:**
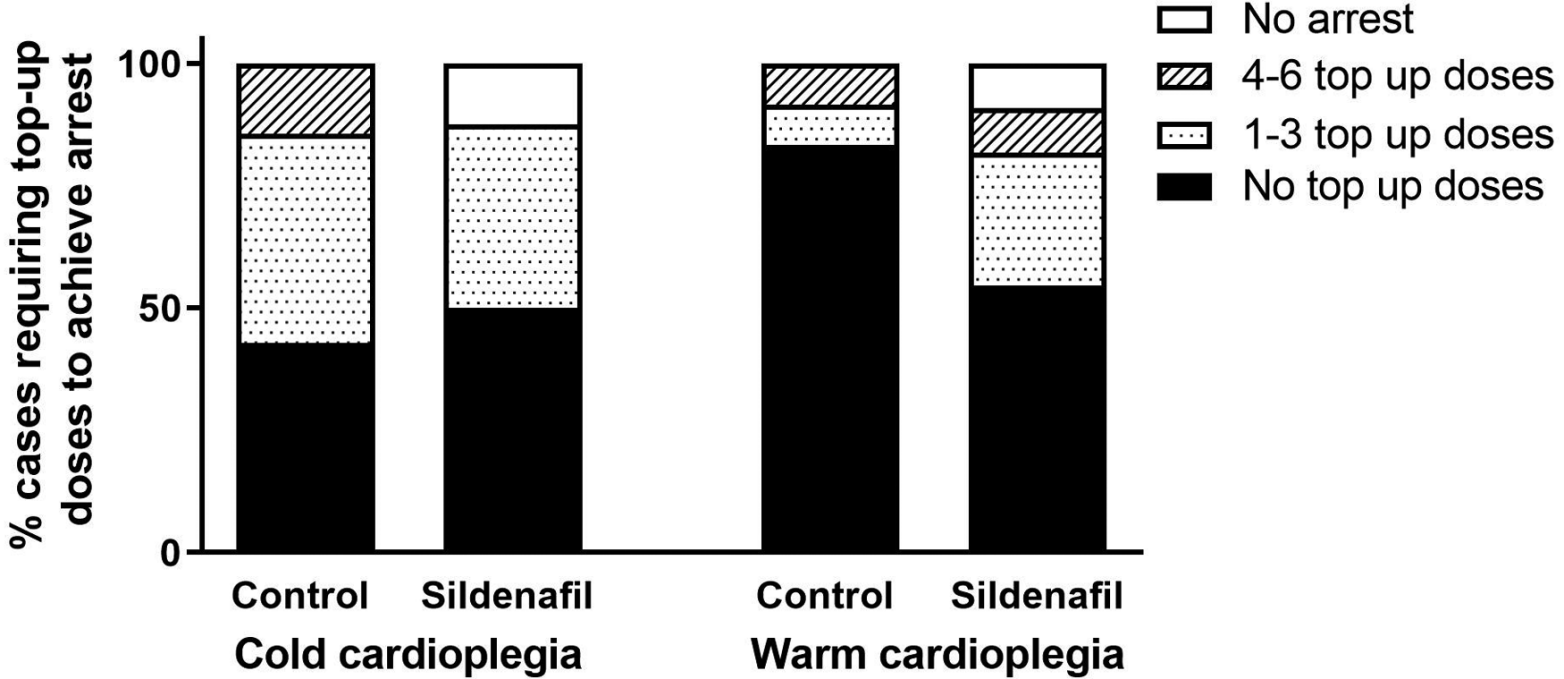
Percent of cases requiring cardioplegic top-up doses in order to achieve cardiac arrest. Data shows the percentage of piglets in each group (cold cardioplegia; control *n* = 7, sildenafil *n* = 8, warm cardioplegia; control *n* = 12, sildenafil *n* = 11) which required no top-up doses (black bar), between 1 and 3 top-up doses (dotted bar), between 4 and 6 top-up doses (hashed bar) or did not achieve arrest (white bar). There were no significant differences between the control and sildenafil groups for either the cold or warm cardioplegia experiments (Chi^2^ test).

Figure 1 displays the percentage of cases in each group which required top-up doses to achieve cardiac arrest. There were no significant differences between the number of top-up doses needed by the control and sildenafil groups for either cold or warm cardioplegia, however with the warm cardioplegia there was a trend for the control group to require less top-up doses (*p* = 0.134). In each of the sildenafil groups there was one case which could not be arrested; these animals are not included in the remainder of the analysis.

**Figure 2:**
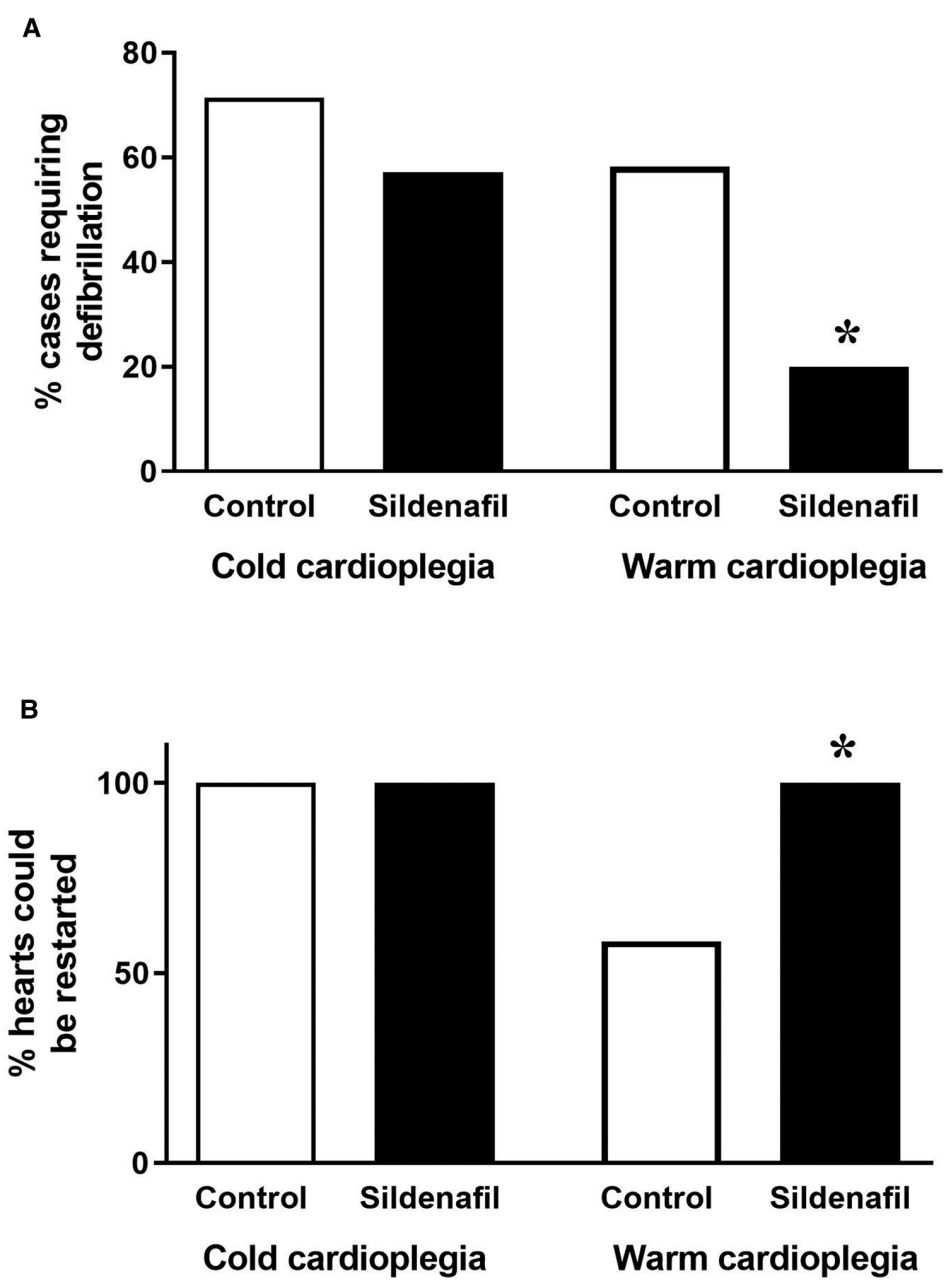
Ease of restarting the hearts. Data shows (**A**) the percentage of cases requiring defibrillation following the removal of the aortic cross-clamp and (**B**) the percentage of hearts which it was possible to restart (cold cardioplegia; control *n* = 7, sildenafil *n* = 7, warm cardioplegia; control *n* = 12, sildenafil *n* = 10). *p < 0.05 compared to control group (Chi^2^ test).

In the warm cardioplegia experiment, significantly less piglets in the sildenafil group required defibrillation to achieve a sinus rhythm following removal of the aortic cross-clamp compared to the control group (Figure 2A). 100% of hearts in the warm sildenafil group were successfully restarted, compared to only 58% in the control group (Figure 2B). With cold cardioplegia however there was no significant difference between the control and sildenafil groups in the proportion of hearts which required defibrillation (Figure 2A) and all hearts were successfully restarted in both groups (Figure 2B).

#### 3.3. Changes in cardiac metabolites during cardioplegic arrest and CPB

**Figure 3:**
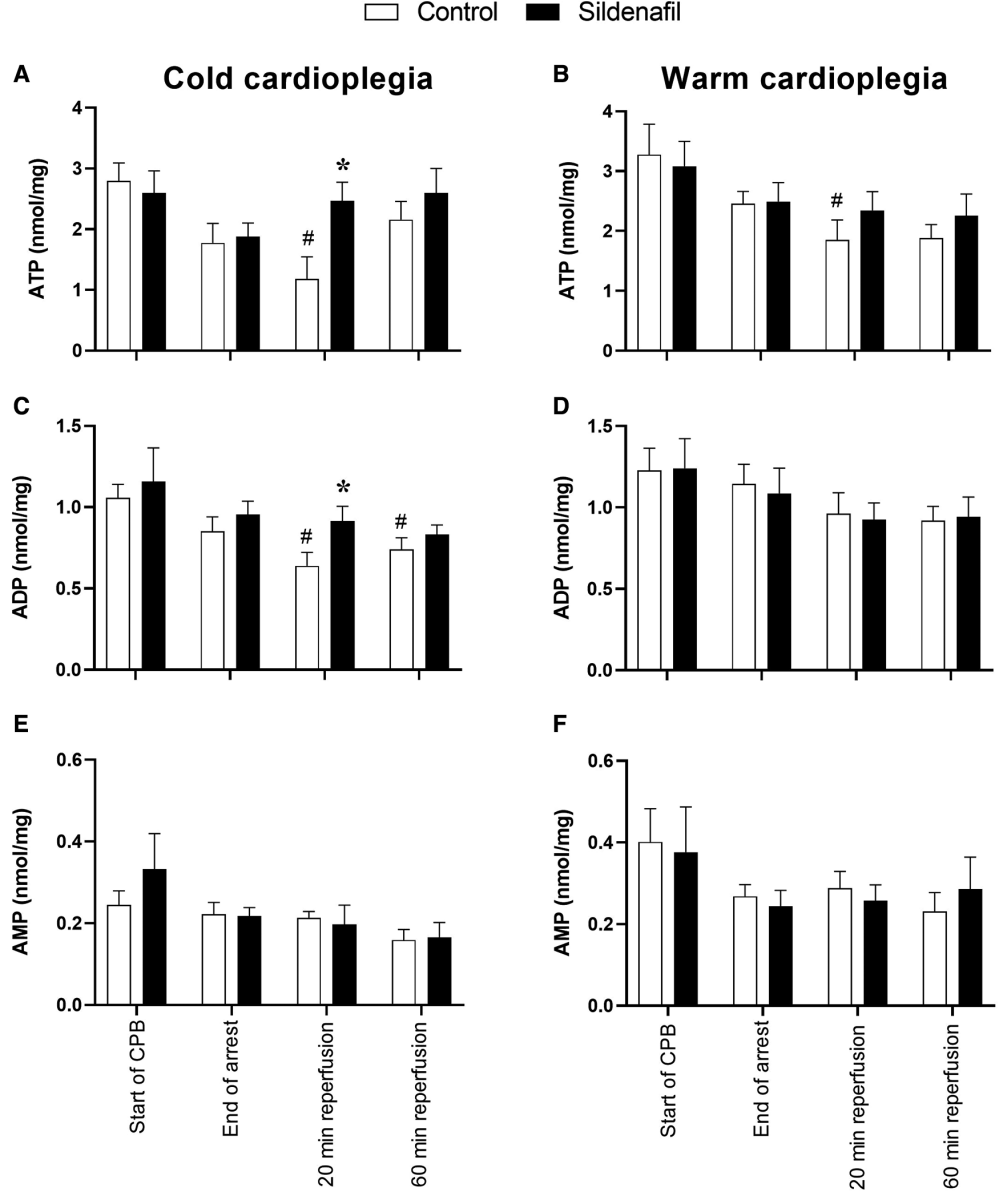
Concentrations of adenine nucleotides in cardiac tissue biopsies. Values are mean ± SEM for metabolite concentrations for control (white bar) and sildenafil treated (black bar) pigs which underwent (**A,C,E**) the cold cardioplegia protocol (*n* = 7 per group) or (**B,D,F**) the warm cardioplegia protocol (control: *n* = 12, sildenafil: *n* = 10). *significant difference vs. corresponding control sample, unpaired two-tailed student's *t*-test or Mann–Whitney *U* test as appropriate. #significant difference vs. the “start of CPB” timepoint in the same treatment group (one-way ANOVA with *post-hoc* Dunnett's test). Two-way mixed model ANOVAs revealed only a significant “between subjects effect” of time for each graph.

The concentration of ATP tended to decrease over the period of cardioplegic arrest, and for both the cold and warm protocols the ATP concentration at 20 min reperfusion was significantly reduced compared to the ATP levels prior to cardioplegic arrest (Figures 3A,B). ATP levels in the cold cardioplegia protocol were significantly higher in the sildenafil group compared to the control group at this timepoint (Figure 3A), suggesting a faster recovery of ATP levels with sildenafil treatment. A similar pattern is seen in the warm sildenafil experiment, however the difference is not significant (Figure 3B). In the cold sildenafil experiment, ADP concentrations follow a similar pattern to the ATP concentrations; in the control group there was significantly reduced ADP concentrations at 20 and 60 min reperfusion compared to before cardioplegic arrest but this was not the case in the sildenafil group (Figure 3C). There was also a significant increase in ADP concentration at 20 min reperfusion in the sildenafil group compared to the control group. For the warm cardioplegia experiment however, the concentrations of ADP are similar between the control and sildenafil groups at each timepoint (Figure 3D). There were no significant differences in AMP levels (Figures 3E,F).

**Figure 4:**
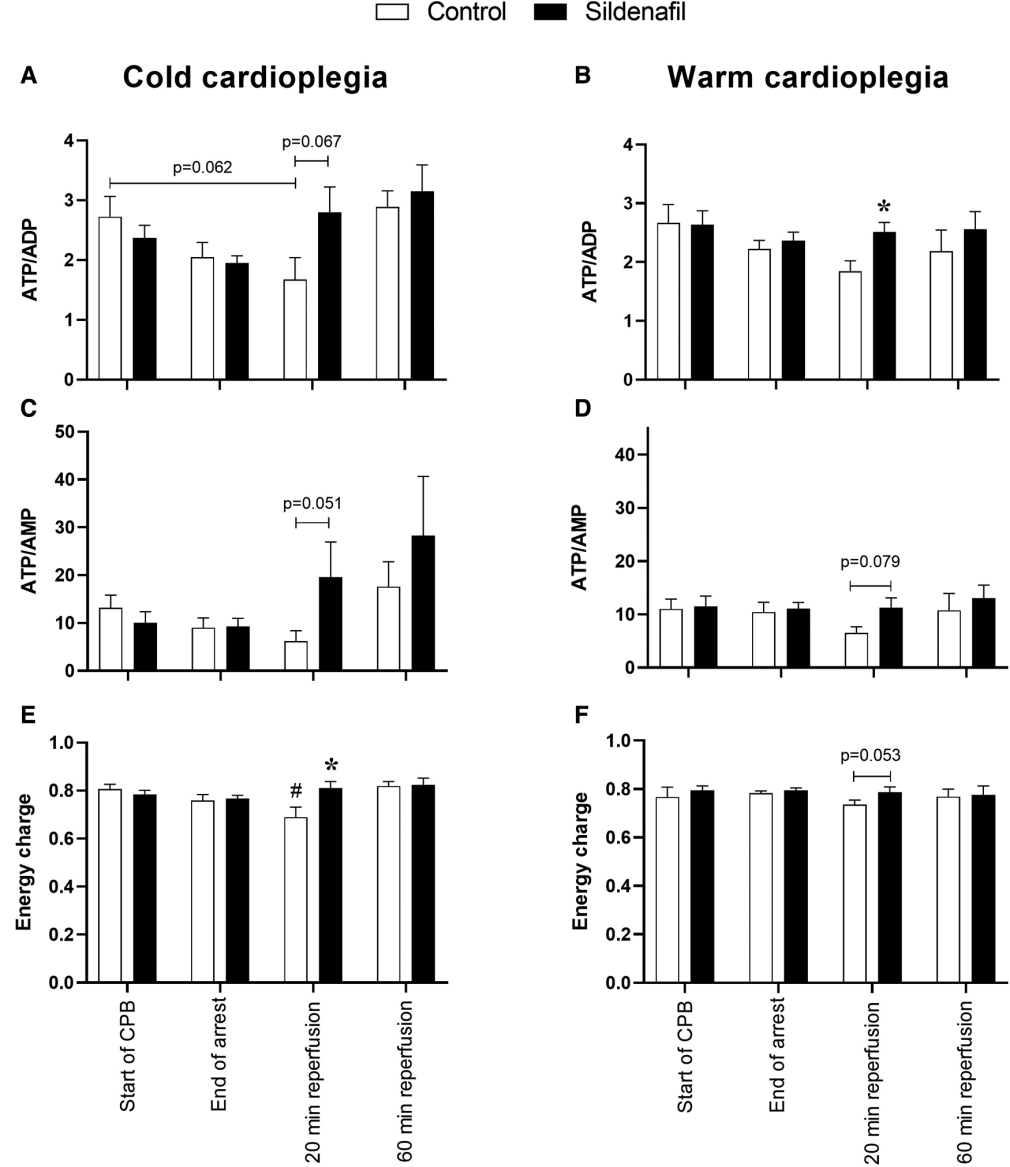
Metabolic ischaemic stress. Values are mean ± SEM for metabolite concentrations for control (white bar) and sildenafil treated (black bar) pigs which underwent (**A,C,E**) the cold cardioplegia protocol (*n* = 7 per group) or (**B,D,F**) the warm cardioplegia protocol (control: *n* = 12, sildenafil: *n* = 10). *significant difference vs. corresponding control sample, unpaired two-tailed student's *t*-test or Mann–Whitney *U* test as appropriate. #significant difference vs. the “start of CPB” timepoint in the same treatment group (one-way ANOVA with *post-hoc* Dunnett's test). Horizontal brackets show trends where the *p*-value was near significance. Two-way mixed model ANOVAs revealed no significant differences except for a significant “between subjects effect” of time for panel A.

Figure 4 displays some ratios calculated from the data in Figure 3, which can be interpreted as markers of ischaemic stress. The ATP/ADP ratio was significantly higher at 20 min reperfusion in the sildenafil group compared to the control group in the warm cardioplegia protocol (Figure 4B). A similar trend existed for the cold cardioplegia protocol (Figure 4A, *p* = 0.067). Both the cold and warm protocols also showed a trend for an increased ratio of ATP/AMP at the 20 min reperfusion timepoint (Figures 4C,D). Energy charge was significantly reduced at 20 min reperfusion compared to before cardioplegic arrest in the control group of the cold cardioplegia protocol (Figure 4E), but was significantly higher in the sildenafil treated group compared to the control group at this timepoint. There was also a trend for increased energy charge with sildenafil treatment at 20 min reperfusion in the warm cardioplegia protocol (Figure 4F), but this did not reach significance (*p* = 0.053).

**Figure 5:**
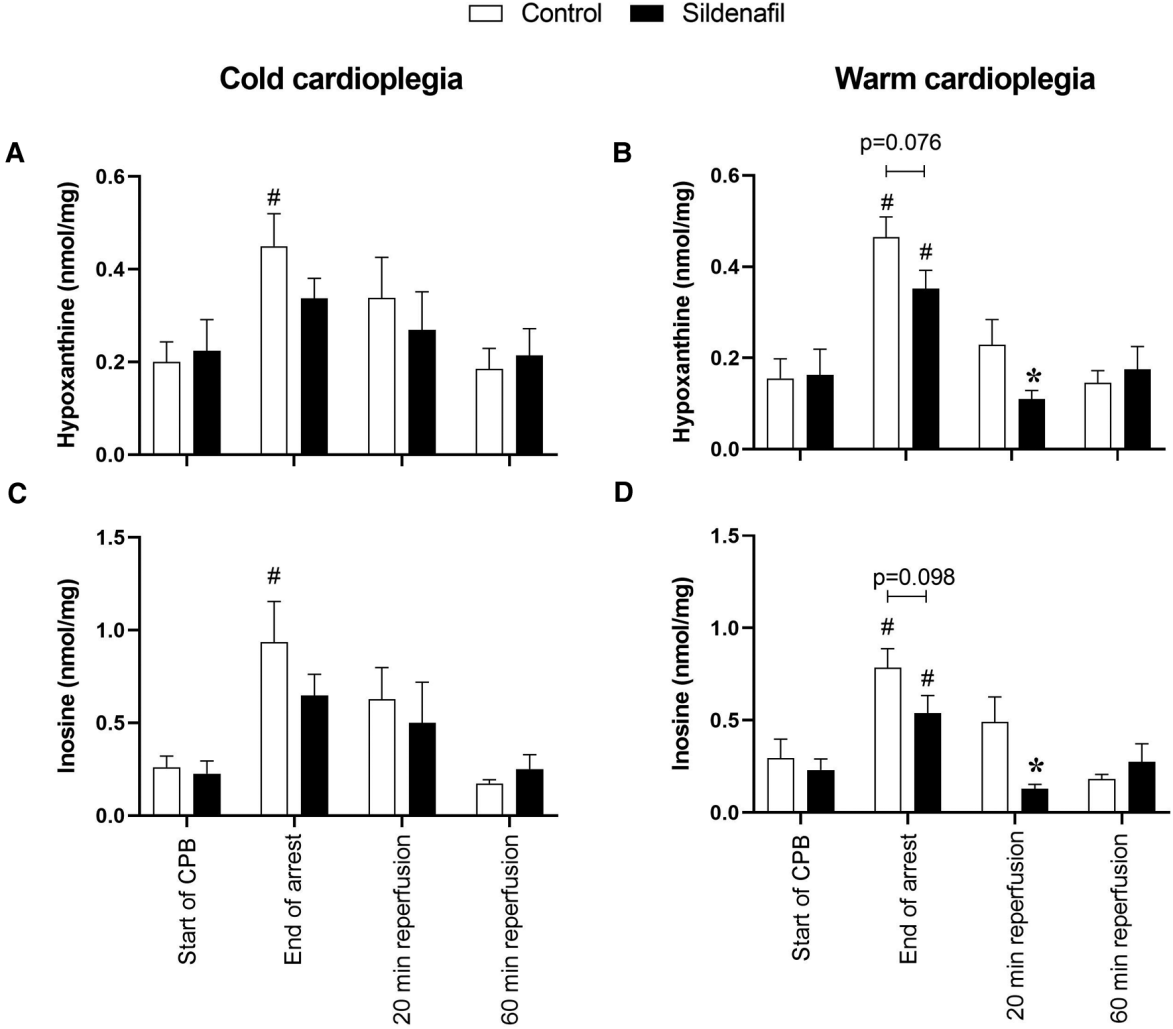
Purine concentrations. Values are mean ± SEM for metabolite concentrations for control (white bar) and sildenafil treated pigs (black bar) which underwent (**A,C**) the cold cardioplegia protocol (*n* = 7 per group) or (**B,D**) the warm cardioplegia protocol (control: *n* = 12, sildenafil: *n* = 10). * significant difference vs. corresponding control sample, unpaired two-tailed student's *t*-test or Mann–Whitney *U* test as appropriate. #significant difference vs. the “start of CPB” timepoint in the same treatment group (one-way ANOVA with *post-hoc* Dunnett's test). Horizontal brackets show trends where the *p*-value was near significance. Two-way mixed model ANOVAs revealed only a significant “between subjects effect” of time for panels (**B–D**), and a significant “between subjects effect” of treatment for panel (**D**).

The concentrations of hypoxanthine and inosine were significantly increased by the end of cardioplegic arrest compared to before arrest in the control groups of both the cold and warm protocols (Figure 5). In the warm cardioplegia protocol the concentrations of these metabolites were also significantly increased compared to baseline in the sildenafil group, but this was not the case with the cold cardioplegia protocol. In the warm cardioplegia protocol both metabolites also showed a significantly reduced concentration in the sildenafil treated group compared to the control group at 20 min reperfusion, suggestive of faster recovery to basal levels.

Concentrations of GTP, GDP, GMP, IMP, adenosine and βNAD are shown in Tables 3 (cold cardioplegia protocol) and 4 (warm cardioplegia protocol). In the cold cardioplegia experiment there was notably a significant increase in GTP concentration at the 20 min reperfusion timepoint in the sildenafil group compared to the control group, suggestive of a faster recovery of GTP to baseline levels with sildenafil treatment (Table 3). In the warm cardioplegia experiment, the concentration of adenosine was significantly increased at the end of cardioplegic arrest compared to the concentration pre-cardioplegic arrest in the sildenafil treatment group, however at 20 min reperfusion the adenosine concentration was significantly lower in the sildenafil group compared to the control group at that timepoint (Table 4).

**Table 3:** Concentration of other ATP-related metabolites in cardiac biopsies from pigs undergoing the cold cardioplegia protocol.

	Start of CPB			End of arrest			20 min reperfusion			60 min reperfusion		
	Control	Sildenafil	*p*-value	Control	Sildenafil	*p*-value	Control	Sildenafil	*p*-value	Control	Sildenafil	*p*-value
GTP	0.11 ± 0.02	0.11 ± 0.02	0.969	0.08 ± 0.02	0.09 ± 0.01	0.684	0.06 ± 0.02	0.12 ± 0.01*	**0** **.** **017**	0.12 ± 0.01	0.12 ± 0.01	0.895
GDP	0.060 ± 0.038	0.023 ± 0.004	0.836	0.023 ± 0.006	0.021 ± 0.003	0.804	0.023 ± 0.010	0.021 ± 0.004	0.628	0.021 ± 0.003	0.021 ± 0.003	0.974
GMP	0.011 ± 0.003	0.007 ± 0.003	0.335	0.006 ± 0.003	0.009 ± 0.003	0.456	0.004 ± 0.002	0.006 ± 0.003	0.731	0.010 ± 0.002	0.013 ± 0.004	0.660
IMP	0.07 ± 0.02	0.04 ± 0.02	0.299	0.03 ± 0.01	0.04 ± 0.01	0.710	0.02 ± 0.01	0.04 ± 0.01	0.135	0.03 ± 0.01	0.05 ± 0.01	0.309
Adenosine	0.65 ± 0.33	0.30 ± 0.06	0.628	0.38 ± 0.12	0.23 ± 0.04	0.383	0.51 ± 0.17	0.30 ± 0.10	0.342	0.20 ± 0.04	0.17 ± 0.03	0.509
*β*NAD	1.02 ± 0.52	0.54 ± 0.08	0.945	0.50 ± 0.07	0.45 ± 0.05	0.710	0.39 ± 0.09	0.54 ± 0.07	0.226	0.48 ± 0.05	0.51 ± 0.04	0.665

Values are mean ± SEM, *n* = 7 per group.

* Significant difference vs. corresponding control sample, unpaired two-tailed student's *t*-test or Mann–Whitney *U* test as appropriate (*p*-values are shown). There were no significant differences between each point vs. the “start of CPB” timepoint in the same treatment group (one-way ANOVA with *post-hoc* Dunnett's test). Two-way mixed model ANOVAs also revealed no significant differences except for a significant “between subjects effect” of time for ATP, ADP and AMP.

**Table 4:** Concentration of other ATP-related metabolites in cardiac biopsies from pigs undergoing the warm cardioplegia protocol.

	Start of CPB			End of arrest			20 min reperfusion			60 min reperfusion		
	Control	Sildenafil	*p*-value	Control	Sildenafil	*p*-value	Control	Sildenafil	*p*-value	Control	Sildenafil	*p*-value
GTP	0.22 ± 0.03	0.21 ± 0.03	0.771	0.15 ± 0.01	0.16 ± 0.02	0.781	0.14 ± 0.02	0.15 ± 0.02	0.632	0.16 ± 0.01	0.16 ± 0.02	0.635
GDP	0.059 ± 0.006	0.060 ± 0.010	0.821	0.052 ± 0.005	0.043 ± 0.006	0.253	0.042 ± 0.004	0.036 ± 0.004	0.351	0.044 ± 0.004	0.042 ± 0.005	0.562
GMP	0.043 ± 0.015	0.051 ± 0.025	0.674	0.041 ± 0.011	0.028 ± 0.004	0.381	0.021 ± 0.003	0.015 ± 0.003	0.172	0.033 ± 0.007	0.042 ± 0.020	0.368
IMP	0.32 ± 0.13	0.35 ± 0.19	0.381	0.07 ± 0.01	0.06 ± 0.01	0.674	0.06 ± 0.01	0.08 ± 0.02	1.000	0.44 ± 0.23	0.17 ± 0.08	0.181
Adenosine	0.033 ± 0.007	0.028 ± 0.007	0.674	0.058 ± 0.006	0.053 ± 0.010**	0.645	0.053 ± 0.015	0.021 ± 0.004*	0.028	0.022 ± 0.007	0.017 ± 0.006	0.313
βNAD	0.57 ± 0.08	0.54 ± 0.09	0.628	0.52 ± 0.04	0.49 ± 0.06	0.596	0.52 ± 0.05	0.51 ± 0.06	0.973	0.46 ± 0.07	0.51 ± 0.08	1.000

Values are mean ± SEM, *n* = 12 (control group) or 10 (sildenafil group).

* Significant difference vs. corresponding control sample, unpaired two-tailed student's *t*-test or Mann–Whitney *U* test as appropriate (*p*-values are shown).

** Significant difference vs. the “start of CPB” timepoint in the same treatment group (one-way ANOVA with *post-hoc* Dunnett's test). Two-way mixed model ANOVAs revealed no significant differences except for a significant “between subjects effect” of time for ATP, ADP and AMP.

#### 3.4. Effect of surgery on early cardiac troponin I release

**Figure 6:**
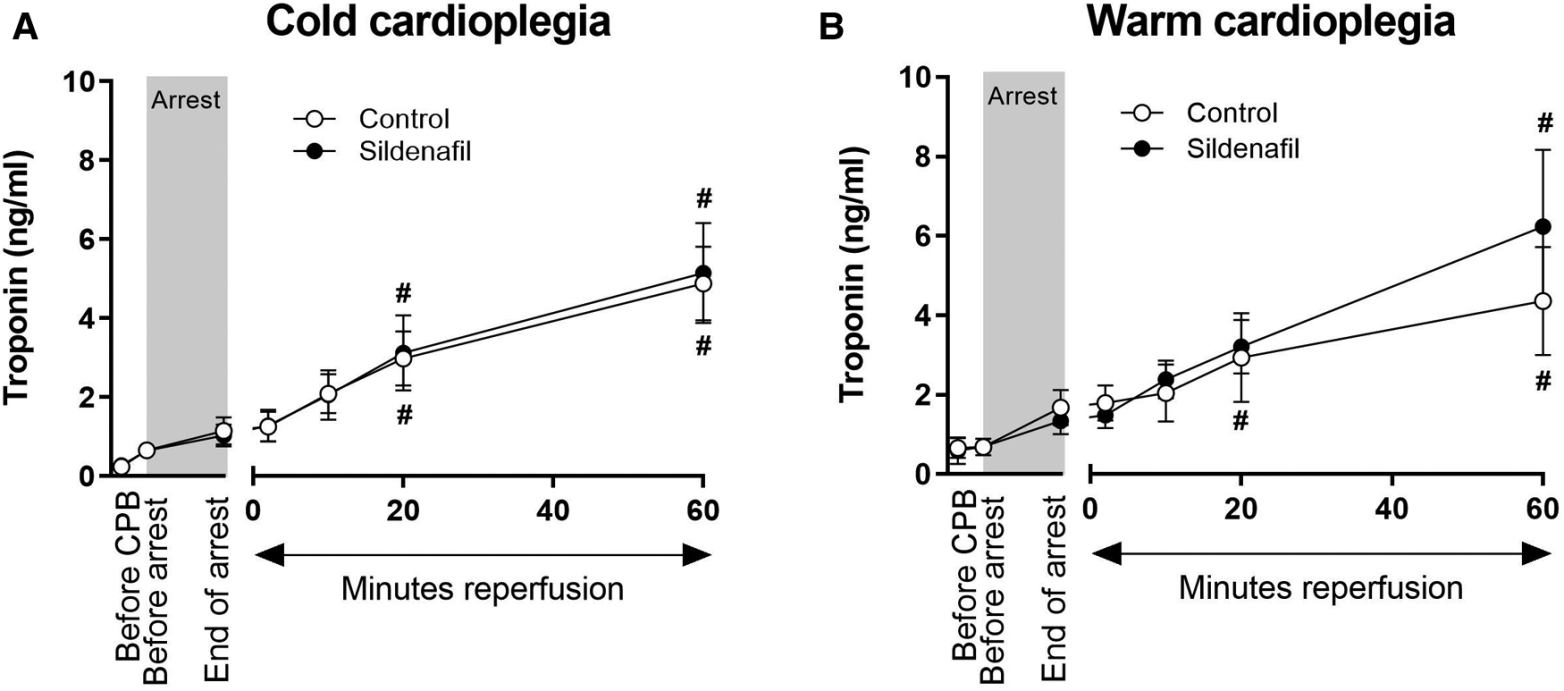
Troponin release. Values are mean ± SEM for plasma troponin concentrations for control (white circles) and sildenafil treated (black circles) pigs whose hearts were arrested with (**A**) cold cardioplegia (control: *n* = 7, sildenafil: *n* = 7) or (**B**) warm cardioplegia (control: *n* = 12, sildenafil: *n* = 9). # represents a significant difference vs. the “Before CPB” timepoint in the same treatment group (one-way ANOVA with *post-hoc* Dunnett's test). For both A and B, there were no significant differences between the control and sildenafil treated groups at any timepoint (unpaired two-tailed student's *t*-test or Mann–Whitney *U* test as appropriate), and two-way mixed model ANOVAs revealed only a significant “between subjects effect” of time.

Figure 6 displays the plasma troponin concentration in all treatment groups. Troponin increased over time in all four treatment groups. There were no significant differences in the amount of troponin released between the control group and the sildenafil treated group for either the cold cardioplegia or the warm cardioplegia protocols.

## 4. Discussion

This study investigated the effect of cardioplegia supplementation with a clinically relevant (10 nM) concentration of the PDE5 inhibitor sildenafil in piglets undergoing CPB surgery. Sildenafil was tested using both the warm and cold cardioplegia protocols currently routinely used in Bristol for paediatric heart surgery. There are some differences between the two protocols for example in the timing of top-up doses and the amount of cardioplegia received by the heart (details in the methods section). For this reason the discussion will not compare the results of the warm and cold protocols, but rather will focus on the effect of addition of sildenafil to each.

### 4.1. Sildenafil and cardioplegic arrest

In our warm cardioplegia protocol, addition of sildenafil to the cardioplegia significantly increased the success rate of being able to successfully wean the hearts from CPB compared to the control group, with the piglets in the sildenafil group also requiring significantly fewer electrical shocks to return to sinus rhythm. Previous investigations into whether sildenafil has a cardiac inotropic effect have proved controversial. Several human studies have found no evidence of an inotropic effect of sildenafil, *in vivo* or *in vitro* (42–44), whilst others have reported that sildenafil administration is associated with increases in cardiac index, haemodynamic changes in the response to exercise and alterations in the concentrations of cGMP and cAMP in the heart (45–47). Studies in animal models have also suggested sildenafil may have inotropic effects under certain conditions, with some studies demonstrating an increase in cardiac inotropy with infusion of sildenafil in anaesthetized animals (48), or a sildenafil induced decrease in the rate of cAMP breakdown (49). Clearly whether sildenafil does cause changes in cardiac contraction is dependent on many factors including the species, dosage, route of administration and any underlying pathology. Interestingly there was no evidence of this when cold cardioplegia was used; it is likely that any inotropic effects were offset by the cold temperature, which is known to depolarize the heart (50). Any inotropic influence of drugs added to cardioplegia (a solution which aims to arrest the heart) is undesirable, and this effect requires further investigation.

### 4.2. Sildenafil and cardiac metabolism

In both the warm and cold cardioplegia protocols, sildenafil had some beneficial effects on cardiac metabolism. For both protocols, ATP levels decreased over the period of ischaemia and early reperfusion, and in both control groups the concentration of ATP at 20 min reperfusion was significantly reduced compared to the basal levels. This is in concordance with previous work which has demonstrated that, upon reperfusion, cardiac function recovers faster than ATP levels (51). There is also a known association between decreased ATP/ADP and ATP/AMP ratios and worse cardiac function (52). Both the warm and cold cardioplegia protocols demonstrated signs of improved ATP metabolism with sildenafil treatment, with a significantly greater ATP concentration at 20 min reperfusion in the sildenafil group compared to the control group of the cold cardioplegia protocol and a significantly improved ATP/ADP ratio at 20 min reperfusion in the sildenafil group of the warm cardioplegia protocol (a similar trend was seen in the cold protocol). Sildenafil treatment in the cold cardioplegia protocol also significantly improved energy charge at 20 min reperfusion, with a similar trend in the warm cardioplegia protocol. Energy charge is calculated from the concentrations of ATP, ADP and AMP and has been found to correlate well with the levels of enzymes involved in ATP utilization—hence it has been described as a measure of metabolic flux (41).

In the control groups of both protocols and the sildenafil group of the warm protocol, there was a significant increase in the concentrations of hypoxanthine and inosine compared to basal levels by the end of ischaemia. Hypoxanthine and inosine are purine catabolites, known to be accumulated during ischaemia and released during reperfusion (53, 54). In the warm cardioplegia protocol, both metabolites were significantly lower compared to their respective control groups at 20 min reperfusion (and similar trends were measurable at the end of ischaemia). Whether this is driven mainly by reduced accumulation during ischaemia or faster release during reperfusion requires more investigation, especially as blockade of the release of the purine metabolites has been linked to cardioprotection (54).

### 4.3. Sildenafil and cardiac injury

Piglets in all treatment groups demonstrated an increase in plasma troponin concentration throughout the experiment, as expected. There is no apparent effect of sildenafil treatment. However, previous experiments of I/R injury in pigs have suggest that troponin levels will continue to rise significantly following this initial 60 min reperfusion period, likely peaking at around 12 h (55, 56). Therefore, our results likely only show a small portion of the troponin response to surgery.

### 4.4. Limitations and future work

The current study did not investigate the mechanisms of action by which sildenafil is cardioprotective. This was due to a limitation in the amount of cardiac tissue that could be collected mid protocol without compromising cardiac function. Further experiments investigating changes in PKG, PKC and NO signalling pathways are required, and direct measurements of cardiac function would also be beneficial. As mentioned above, the study was also limited by the short reperfusion period. Use of a longer reperfusion period or even recovery of the animals would be beneficial to investigate the longer-term effects of cardioplegia supplementation with sildenafil. Another limitation is that these are healthy pigs, whilst cardiac surgery is never performed on a healthy heart. Study of how PDE-5 expression is affected by different pathologies and testing of sildenafil in an animal model with a diseased heart would be highly beneficial. Dose response curves and testing of the timing of sildenafil administration would also be informative—for example it is possible that sildenafil may be more effective if given prior to surgery. Another limitation is the fact that only female pigs were used, meaning that sex was controlled for but not investigated. Further study in male pigs is required to investigate any intersex differences.

## 5. Conclusions

This study demonstrated that supplementation of cardioplegia with sildenafil during piglet CPB surgery has beneficial metabolic effects as shown by improving the levels of energy rich phosphates upon reperfusion. This improved metabolic status was associated with improved ability to wean off bypass. More work is required to investigate the mechanisms of action, the longer -term effects and to optimize the protocol for sildenafil administration. Cardioprotection for paediatric patients is currently not as effective as in adult patients (10–14) and for a long time has been described as inadequate (57–59). Therefore, these findings may represent an exciting step towards improving cardioprotection for this highly vulnerable group of patients.

## Data Availability

The raw data supporting the conclusions of this article will be made available by the authors, without undue reservation.
