# Supplementary material for: The effect of cardioplegic supplementation with sildenafil on cardiac energetics in a piglet model of cardiopulmonary bypass and cardioplegic arrest with warm or cold cardioplegia
Source: Front Cardiovasc Med. 2023 Jun 7;10:1194645. doi: 10.3389/fcvm.2023.1194645 (PMC10282544; doi:10.3389/fcvm.2023.1194645)
Supplement: Supplementary file 1 [file Datasheet1.docx]

Supplementary Material

**Table S1 Blood gases for the cold cardioplegia protocol.** Values are mean ± SEM, n=7 per group. ^#^ represents a significant difference *vs* the ‘Before CPB’ value in the same treatment group (one-way ANOVA with *post-hoc* Dunnett’s test). There were no significant differences between the sildenafil group and the control group at any timepoint (unpaired students t-test or Mann-Whitney U test as appropriate). CPB= cardiopulmonary bypass, AoX = aortic cross-clamp, pCO_2_= partial pressure of carbon dioxide, pO_2_= partial pressure of oxygen, cHCO_3_^-^ = actual bicarbonate, cTCO_2_ = total plasma carbon dioxide concentration, BUN = blood urea nitrogen.

|  | **Before CPB** | | **AoX on** | | **After AoX release** | |
| --- | --- | --- | --- | --- | --- | --- |
|  | **Control** | **Sildenafil** | **Control** | **Sildenafil** | **Control** | **Sildenafil** |
| **pH** | 7.42 ± 0.02 | 7.40 ± 0.03 | 7.49 ± 0.02 | 7.45 ± 0.03 | 7.34 ± 0.02 | 7.32 ± 0.06 |
| **pCO_2_ (mmHg)** | 47.83 ± 3.12 | 51.28 ± 1.81 | 34.98 ± 4.05 | 39.06 ± 3.86 | 48.70 ± 1.74 | 51.16 ± 5.35 |
| **pO_2_ (mmHg)** | 397.30 ± 95.18 | 299.58 ± 86.63 | 324.31 ± 50.52 | 276.99 ± 45.43 | 232.72 ± 33.01 | 229.94 ± 34.79 |
| **cHCO_3_^-^ (mM)** | 31.07 ± 0.58 | 31.56 ± 1.75 | 25.84 ± 2.32 | 26.97 ± 1.81 | 24.05 ± 2.37 | 25.94 ± 2.11 |
| **Base excess (mM)** | 6.63 ± 0.22 | 7.02 ± 2.12 | 2.46 ± 2.13 | 3.06 ± 1.78 | 0.29 ± 1.16 | -0.02 ± 2.69 |
| **Oxygen saturation (%)** | 99.93 ± 0.07 | 99.87 ± 0.19 | 99.87 ± 0.08 | 98.04 ± 1.75 | 98.81 ± 0.71 | 99.48 ± 0.36 |
| **Na^2+^ (mM)** | 142.67 ± 0.88 | 142.00 ± 1.00 | 137.57 ± 1.11^#^ | 137.81 ± 0.67^#^ | 138.17 ± 0.90^#^ | 137.20 ± 0.58^#^ |
| **K^+^ (mM)** | 3.73 ± 0.13 | 3.82 ± 0.27 | 4.75 ± 0.11^#^ | 4.72 ± 0.22^#^ | 4.54 ± 0.25^#^ | 4.48 ± 0.15 |
| **Ca^2+^ (mM)** | 1.48 ± 0.01 | 1.47 ± 0.05 | 1.34 ± 0.04 | 1.37 ± 0.04 | 1.43 ± 0.03 | 1.45 ± 0.09 |
| **Cl^-^ (mM)** | 103.33 ± 1.20 | 102.20 ± 1.62 | 99.58 ± 1.11 | 100.36 ± 1.97 | 101.13 ± 1.85 | 99.90 ± 0.68 |
| **cTCO_2_ (mM)** | 30.87 ± 0.54 | 31.25 ± 0.15 | 24.79 ± 3.07 | 69.95 ± 39.45 | 25.52 ± 0.29 | 24.83 ± 0.90 |
| **Anion gap (mM)** | 13.33 ± 1.20 | 14.00 ± 1.00 | 18.40 ± 2.14 | 18.00 ± 4.00 | 16.50 ± 1.26 | 17.00 ± 1.00 |
| **Haematocrit (%)** | 21.33 ± 1.20 | 22.20 ± 0.86 | 16.29 ± 1.31 | 15.62 ± 1.55^#^ | 17.77 ± 0.85 | 15.50 ± 1.41^#^ |
| **Haemoglobin (g/dL)** | 7.30 ± 0.46 | 7.62 ± 0.32 | 5.08 ± 0.44^#^ | 5.30 ± 0.52^#^ | 6.09 ± 0.40 | 5.38 ± 0.45^#^ |
| **Glucose (mM)** | 3.53 ± 0.48 | 4.56 ± 0.71 | 8.48 ± 1.14 | 7.91 ± 0.80 | 12.77 ± 1.96^#^ | 13.13 ± 3.48^#^ |
| **Lactate (mM)** | 1.10 ± 0.17 | 1.59 ± 0.28 | 3.72 ± 0.47^#^ | 3.62 ± 0.38^#^ | 6.24 ± 0.77^#^ | 5.70 ± 0.74^#^ |
| **BUN (mg/dL)** | 7.00 ± 1.00 | 6.80 ± 0.58 | 6.90 ± 1.09 | 5.99 ± 0.43 | 9.08 ± 1.16 | 7.40 ± 0.93 |
| **Creatinine (µM)** | 110.67 ± 20.61 | 94.60 ± 17.05 | 122.70 ± 11.69 | 95.71 ± 11.90 | 114.67 ± 16.20 | 102.50 ± 12.84 |

**Table S2 Blood gases for the warm cardioplegia protocol.** Values are mean ± SEM, n=11 (control group) or 10 (sildenafil group). * represents a significant difference compared to control group (p<0.05, unpaired students t-test or Mann-Whitney U test as appropriate). ^#^ represents a significant difference *vs* the ‘Before CPB’ value in the same treatment group (one-way ANOVA with *post-hoc* Dunnett’s test)). CPB= cardiopulmonary bypass, AoX = aortic cross-clamp, pCO_2_= partial pressure of carbon dioxide, pO_2_= partial pressure of oxygen, cHCO_3_^-^ = actual bicarbonate, cTCO_2_ = total plasma carbon dioxide concentration.

|  | **Before CPB** | | **AoX on** | | **After AoX release** | |
| --- | --- | --- | --- | --- | --- | --- |
|  | **Control** | **Sildenafil** | **Control** | **Sildenafil** | **Control** | **Sildenafil** |
| **pH** | 7.37 ± 0.05 | 7.43 ± 0.03 | 7.46 ± 0.04 | 7.49 ± 0.02 | 7.35 ± 0.06 | 7.41 ± 0.04 |
| **pCO_2_ (mmHg)** | 54.27 ± 8.15 | 48.57 ± 3.31 | 40.40 ± 3.79 | 36.73 ± 2.42^#^ | 43.64 ± 6.28 | 39.51 ± 3.84 |
| **pO_2_ (mmHg)** | 353.43 ± 25.19 | 355.27 ± 44.61 | 213.69 ± 25.22^#^ | 215.92 ± 35.30^#^ | 187.08 ± 47.17^#^ | 196.28 ± 25.60^#^ |
| **cHCO_3_^-^ (mM)** | 30.32 ± 1.65 | 31.89 ± 0.98 | 27.70 ± 0.88 | 27.40 ± 0.69^#^ | 23.64 ± 1.55^#^ | 24.19 ± 0.88^#^ |
| **Base excess (mM)** | 5.10 ± 1.78 | 7.57 ± 1.18 | 3.82 ± 1.13 | 4.04 ± 0.60^#^ | -1.34 ± 2.54 | -0.51 ± 1.06^#^ |
| **Oxygen saturation (%)** | 99.95 ± 0.02 | 99.91 ± 0.04 | 99.53 ± 0.18 | 99.33 ± 0.29 | 89.74 ± 9.89 | 99.01 ± 0.46 |
| **Na^2+^ (mM)** | 144.50 ± 2.14 | 141.36 ± 1.19 | 139.14 ± 1.11^#^ | 139.61 ± 1.04 | 139.00 ± 0.45 | 140.36 ± 0.59 |
| **K^+^ (mM)** | 3.18 ± 0.27 | 3.59 ± 0.09 | 4.83 ± 0.22^#^ | 5.28 ± 0.47^#^ | 5.44 ± 0.37^#^ | 4.47 ± 0.13* |
| **Ca^2+^ (mM)** | 1.40 ± 0.10 | 1.50 ± 0.08 | 1.32 ± 0.03 | 1.50 ± 0.05* | 1.46 ± 0.09 | 1.55 ± 0.08 |
| **Cl^-^ (mM)** | 107.50 ± 2.74 | 101.64 ± 1.04 | 102.44 ± 0.87 | 103.25 ± 1.64 | 105.00 ± 1.05 | 102.64 ± 1.05 |
| **cTCO_2_ (mM)** | 32.00 ± 1.77 | 33.39 ± 1.00 | 28.67 ± 0.97 | 28.20 ± 0.74^#^ | 24.98 ± 1.58^#^ | 25.44 ± 0.91^#^ |
| **Anion gap (mM)** | 9.67 ± 0.49 | 11.43 ± 1.11 | 15.06 ± 0.75^#^ | 15.19 ± 0.71^#^ | 15.80 ± 1.39^#^ | 17.86 ± 1.40^#^ |
| **Haematocrit (%)** | 21.00 ± 1.67 | 22.36 ± 1.27 | 13.64 ± 0.80^#^ | 14.89 ± 0.73^#^ | 17.20 ± 1.32 | 14.36 ± 0.73^#^ |
| **Haemoglobin (g/dL)** | 7.13 ± 0.59 | 7.64 ± 0.39 | 4.50 ± 0.28^#^ | 4.93 ± 0.29^#^ | 5.88 ± 0.48 | 4.89 ± 0.25^#^ |
| **Glucose (mM)** | 5.67 ± 0.74 | 5.23 ± 0.75 | 7.97 ± 0.93 | 9.49 ± 0.89^#^ | 15.72 ± 0.92^#^ | 13.48 ± 1.32^#^ |
| **Lactate (mM)** | 1.02 ± 0.26 | 1.94 ± 0.43 | 3.69 ± 0.45^#^ | 4.20 ± 0.56^#^ | 7.71 ± 0.76^#^ | 7.95 ± 0.87^#^ |
| **Creatinine (µM)** | 118.00 ± 19.21 | 128.14 ± 12.17 | 114.06 ± 8.92 | 118.19 ± 5.81 | 140.60 ± 14.79 | 115.79 ± 11.81 |
